# Characterizing temporal genomic heterogeneity in pediatric high-grade gliomas

**DOI:** 10.1186/s40478-017-0479-8

**Published:** 2017-10-30

**Authors:** Ralph Salloum, Melissa K. McConechy, Leonie G. Mikael, Christine Fuller, Rachid Drissi, Mariko DeWire, Hamid Nikbakht, Nicolas De Jay, Xiaodan Yang, Daniel Boue, Lionel M. L. Chow, Jonathan L. Finlay, Tenzin Gayden, Jason Karamchandani, Trent R. Hummel, Randal Olshefski, Diana S. Osorio, Charles Stevenson, Claudia L. Kleinman, Jacek Majewski, Maryam Fouladi, Nada Jabado

**Affiliations:** 1 Brain Tumor Center, Cincinnati Children’s Hospital Medical Center, 3333 Burnet Avenue, Cincinnati, OH 45229 USA; 2 Department of Human Genetics, McGill University, Montreal, QC, H3A 1B1 Canada; 3 Department of Pediatrics, McGill University and McGill University Heath Centre Research Institute, Montreal, QC, H4A 3J1 Canada; 4 Department of Laboratory Medicine and Pathology, Nationwide Children’s Hospital, and the Ohio State University, Columbus, OH 43205 USA; 5 Division of Hematology/Oncology and Bone Marrow Transplantation, Nationwide Children’s Hospital, Columbus, OH 43205 USA; 6 Department of Pathology, Montreal Neurological Hospital, McGill University, Montreal, QC, H3A 2B4 Canada; 7 The Lady Davis Institute, Jewish General Hospital, Montreal, QC, H3T 1E2 Canada

**Keywords:** Pediatric high-grade gliomas, Recurrence, Genomics, Histone 3, *ATRX*, *IDH1*, *NF1*, Tumor evolution

## Abstract

Pediatric high-grade gliomas (pHGGs) are aggressive neoplasms representing approximately 20% of brain tumors in children. Current therapies offer limited disease control, and patients have a poor prognosis. Empiric use of targeted therapy, especially at progression, is increasingly practiced despite a paucity of data regarding temporal and therapy-driven genomic evolution in pHGGs. To study the genetic landscape of pHGGs at recurrence, we performed whole exome and methylation analyses on matched primary and recurrent pHGGs from 16 patients. Tumor mutational profiles identified three distinct subgroups. Group 1 (*n* = 7) harbored known hotspot mutations in Histone 3 (H3) (K27M or G34V) or *IDH1 (*H3/IDH1 mutants) and co-occurring *TP53* or *ACVR1* mutations in tumor pairs across the disease course. Group 2 (*n* = 7), H3/IDH1 wildtype tumor pairs, harbored novel mutations in chromatin modifiers (*ZMYND11, EP300 n* = 2), all associated with *TP53* alterations, or had *BRAF* V600E mutations (*n* = 2) conserved across tumor pairs. Group 3 included 2 tumors with *NF1* germline mutations. Pairs from primary and relapsed pHGG samples clustered within the same DNA methylation subgroup. *ATRX* mutations were clonal and retained in H3G34V and H3/IDH1 wildtype tumors, while different genetic alterations in this gene were observed at diagnosis and recurrence in IDH1 mutant tumors. Mutations in putative drug targets (*EGFR, ERBB2, PDGFRA, PI3K*) were not always shared between primary and recurrence samples, indicating evolution during progression. Our findings indicate that specific key driver mutations in pHGGs are conserved at recurrence and are prime targets for therapeutic development and clinical trials (e.g. H3 post-translational modifications, *IDH1*, *BRAF* V600E). Other actionable mutations are acquired or lost, indicating that re-biopsy at recurrence will provide better guidance for effective targeted therapy of pHGGs.

**Electronic supplementary material:**

The online version of this article (10.1186/s40478-017-0479-8) contains supplementary material, which is available to authorized users.

## Introduction

Genetic and epigenetic molecular profiling techniques have revolutionized our understanding of the etiology and biology of pediatric high-grade gliomas (pHGGs) (reviewed in [20]). Unfortunately, this has not yet led to an improvement in outcome for children with this disease [40] despite the use of agents that target pathways identified through these biological advances. Novel agents for the treatment of pHGGs are first tested in the relapse setting, and target genomic alterations typically present in therapy-naïve diagnostic tumor samples or models. However, there is limited data on the relevance of genomic aberrations at diagnosis on disease progression after multimodal therapy, making the effectiveness of this approach questionable. An improved understanding of temporal and therapy-driven evolution of recurrent pHGGs is therefore needed, especially in the context of hemispheric HGGs that show increased genetic heterogeneity [5, 12, 13, 19, 37, 50, 51].

Clonal evolution is a dynamic process that has been reported in many cancer types [3, 28, 39, 48], even without exposure to therapy [11]. Morrissy et al., have recently demonstrated poor overlap in genetic events between primary and post-treatment medulloblastoma both in murine models and human samples [28]. This included a marked divergence in actionable genes between diagnosis and recurrence, despite conservation of molecular subgroup affiliation [28, 36, 47]. Whole exome sequencing (WES) of 23 initial and recurrent gliomas in adults by Johnson et al., revealed variable genetic relatedness across pairs; in 10 cases, most mutations from diagnosis were not conserved in the recurrent sample, including the *BRAF* V600E hotspot mutation [19]. In adult glioblastoma multiforme (GBM), a longitudinal study of the genetic landscape of 114 untreated and recurrent paired tumors revealed a switch in expression-based subtypes in 63% of cases. Enrichment of a hypermutated phenotype in recurrent disease exposed to temozolomide (TMZ) was also identified, suggesting the occurrence of therapy-induced mutagenesis [45]. Moreover, an analysis of tumor phylogeny revealed that dominant clones at recurrence were infrequently direct descendants of dominant clones from diagnosis [45]. We have previously shown that disease-defining somatic mutations in oncohistones [K27M in Histone 3 (H3) variants (*H3F3A*, *HIST1H3B*)] are spatially stable in diffuse intrinsic pontine glioma (DIPG), and co-occur with highly conserved partners throughout geographically distinct tumor sites [18, 30]. However, limited data on disease recurrence are available for supratentorial pHGGs. This is of major therapeutic interest as hemispheric pHGGs show more genetic variability at diagnosis than midline tumors, the vast majority of which are defined by H3K27M mutations (> 90%) [14, 51]. In the current study, we characterize the temporal genomic heterogeneity in pHGGs by assessing the mutational profile and methylome of paired primary and recurrent tumors with emphasis on supratentorial pHGGs.

## Materials and methods

### Clinical cohort

Institutional review board approval was obtained to perform this retrospective study at Cincinnati Children’s Hospital Medical Center (CCHMC, Study ID: 2014-6849) and Nationwide Children’s Hospital (NCH: IRB15-00143). The patient cohort was chosen based on the availability of material from both the primary and recurrent tumor for each case with a confirmed HGG diagnosis Two neuropathologists (CF and JK) independently reviewed tumor samples. Patient tumor samples were acquired from diagnosis as well as recurrence or autopsy and preserved either as fresh-frozen or formalin fixed paraffin embedded (FFPE) tissue. Blood or other matched normal tissue was obtained when available for germline analysis. To ensure adequate tumor content, hematoxylin and eosin (H&E) slides were reviewed from each frozen specimen, the initial cut of each FFPE block, and an additional cut of FFPE block after scrolls were obtained for DNA extraction. All patient tumor and matched blood samples were collected after informed consent was provided by patients or legal guardians through institutional review board approved protocols at the respective institutions.

### DNA extraction

DNA extraction was carried out from frozen tissue using the Qiagen AllPrep DNA/RNA/miRNA Universal Kit following the manufacturer’s instructions. DNA from FFPE scrolls or core punches were isolated by suspending the paraffin scrolls in deparaffinization solution (Qiagen) followed by DNA extraction using the QIAamp DNA FFPE Tissue Kit. DNA quantification was conducted using the Quant-iT Picogreen dsDNA assay kit (Thermo Fisher Scientific). Droplet digital PCR (ddPCR) assays for H3K27M mutations were performed as previously described [30].

### Whole Exome Sequencing (WES) analysis

The Nextera Rapid Capture Exome kit (Illumina) was used to prepare 36 libraries, and the Agilent SureSelect Reagent Exome kit (Agilent) was used to prepare 6 libraries according to the manufacturer’s instructions. Genomic DNA was extracted from frozen tissue and FFPE blocks representing tumor or normal tissue and from monocytes. Sequencing was performed on the Illumina HiSeq 2000 using rapid-run mode with 100 bp paired-end reads. Adaptor sequences were removed, and reads trimmed for quality using the FASTX-Toolkit (http://hannonlab.cshl.edu/fastx_toolkit/). An in-house program was used to ensure the presence of exclusively paired-reads. We next aligned the reads using Burrows-Wheeler Aligner (BWA) 0.7.7 to GRC37/hg19 as a reference genome. Indel realignment was performed using the Genome Analysis Toolkit (GATK) 29 (https://software.broadinstitute.org/gatk/). Duplicate reads were marked using Picard (http://broadinstitute.github.io/picard/), and excluded from further analyses. The average coverage for all the samples was 69X. Single Nucleotide Variants (SNVs) and short indels were called using our in-house pipeline that exploits three different variant callers: FreeBayes 1.1.0 (https://arxiv.org/abs/1207.3907), SAMtools 1.3.1 (http://samtools.sourceforge.net/) and GATK HaplotypeCaller 3.7 [43]. Thresholds were set for calling a true variant to two out of three variant callers. Next, variants were filtered for quality so at least 10% of reads supported each variant call. ANNOVAR [46] and in-house programs were used to annotate variants that affect protein-coding sequence. Variants were screened to assess whether they had previously been observed in public datasets including the 1000 Genomes Project data set (November 2011), the National Heart, Lung and Blood Institute (NHLBI) Grand Opportunity (GO) exomes as well as in over 3000 exomes previously sequenced at our center (including cancer and non-cancer samples).

### Somatic and putative somatic mutation identification from Whole Exome Sequencing

Protein coding variants were identified as nonsynonymous missense, frameshifts, stopgains, indels, splice variants, and present with a minimum of 5% mutant allele frequency (MAF) or greater. A MAF of > 5% was used in order to reduce removal of low frequency mutations in genes of interest (*H3F3A, TP53, ATRX*, *ZMYND11, LZTR1*). All other reported mutations have a MAF > 15%. To be considered somatic, zero mutant variant reads were present in the normal sample. In some cases where < 2 variant reads were present in the normal, the sequencing alignments were manually checked to verify as sequencing artifacts. Additionally, all somatic and putative somatic variants were manually checked for alignment and sequencing artifacts. To further remove germline SNPs where normal was not available, all samples were filtered based on a MAF (mutant allele frequency) < 0.0005 in the 1000 Genomes Project (November 2011), EVS, and < 0.00005 from ExAC databases. Finally, in cases with no associated normal, variants were only reported when present in COSMIC or high/medium functional impact as assessed using MutationAssessor (http://mutationassessor.org/r3/). Targeted validations on selected variants were performed using Sanger sequencing or high-depth sequencing on an Illumina Miseq as previously described [30].

### Allelic Imbalance and CNV analysis

Allelic Imbalance was assessed in whole exome sequencing data using the ExomeAI program [29] for 15 pHGG tumor pairs using the default parameters of the program. HGG1 was not analyzed due to low WES coverage of the recurrence tumor. Copy number variations (CNVs) were analyzed in 8 pHGG tumor-normal pairs using an in-house program (CNAXX; unpublished) we developed that takes both coverage (normalized average) and the deviation of B allele frequency from 50% into account (adapted from methods used in FishingCNV [38] and ExomeAI [29]). Different CNV events (amplification, deletion and copy neutral LOH) were called based upon the status of the normalized coverage and the B allelic imbalance as we described previously in [30]. We assessed the CNV events at both the chromosomal arm level and the union of the segments called by each of the two features used by the program (i.e. B allele frequency and normalized average coverage). Paired t-tests were performed using the statistical program R version 3.1.1.

### DNA methylation analysis

Illumina 450 K methylation chips were used on 28 samples, and profiling data was analyzed as previously described [14, 32]. In some cases, only the tumor from the primary (HGG1, HGG3, HGG11) or recurrence (HGG12) were included in the final analysis due to insufficient or poor quality FFPE DNA from the respective tumor pair. The raw data were subject to quality control and preprocessing utilizing the R package minfi, and normalized for technical variation between the Infinium I and II probes using the SWAN method. We removed probes on sex chromosomes (chr X, Y), those containing SNPs (dbSNP: https://www.ncbi.nlm.nih.gov/projects/SNP/) as well as non-specific probes that bind to multiple genomic locations. Unsupervised hierarchical clustering was performed using average linkage, and Pearson rank correlation distance on the top 3000 most variable probes selected based on standard deviation of beta values (β-values).

### Immunohistochemistry for MMR proteins

Immunohistochemistry (IHC) staining for expression of MMR proteins (MLH1, MSH2, MSH6, PMS2) was performed on slides cut from FFPE blocks of pHGG samples using conventional methods [53].

## Results

### Patient characteristics

Paired primary and recurrent pHGG tumor samples were available from 16 patients. Median age at diagnosis was 15 years (range: 4–29 years). Among the 16 cases, one patient with a germline *NF1* mutation was initially diagnosed with a WHO grade II glioma with pilocytic features (HGG16) before recurring as a HGG. Median time to progression was 13 months (range: 4–45 months). Fourteen patients received radiation therapy at diagnosis, and 10 patients (63%) had received temozolomide (TMZ) prior to last progression. Five tumors (31%) were midline [2 pontine tumors (HGG2, HGG4), 1 thalamic tumor (HGG8), 2 spinal tumors (HGG1, HGG3)], and 11 tumors (69%) were hemispheric. Two patients (HGG15 and HGG16) were previously known to have germline neurofibromatosis type 1 (NF1). All clinicopathological features are shown in Table 1.

**Table 1:** Clinicopathological features of 16 pediatric High-Grade Glioma (pHGG) primary-recurrence tumor pairs analyzed in this study

Molecular Group	HGG case	Age/sex	Primary (P) Dx	Recurrence (R) Dx	Time to progression (months)	Interval between P and R (months)	Neuro-anatomical Location	Surgery#1	Surgery#2	Germline Available	Treatments between diagnosis and recurrence sample
H3/IDH1 mutant	1	14yo/M	GBM	GBM	8.7	16.2	Midline (Spine)	GTR	NA (autopsy)	Yes	RT+ TMZ, TMZ
	2	18yo/F	AA	GBM	10.3	14.4	Midline (Pons)	STR	NA (autopsy)	Yes	RT+ TMZ, TMZ, bevacizumab, vorinostat
	3	13yo/F	GBM	GBM	6.5	6.5	Midline (Spine)	STR	NA (autopsy)	Yes	RT+ TMZ, rapamycin
	4	4yo/F	GBM	GBM	23.5	23.5	Midline (Pons)	Biopsy	NA (autopsy)	No	RT + vandatenib, dasatinib
	5	12yo/M	GBM	GBM	7.1	8.7	Hemisphere	GTR	NA (autopsy)	No	RT + TMZ, TMZ + bevacizumab
	6	29yo/F	AA focal GBM	GBM	45.2	45.2	Hemisphere	STR	STR	No	RT + TMZ, TMZ
	7	19yo/F	AA	AA	14.3	14.3	Hemisphere	GTR	GTR	No	Surgery
H3/IDH1 wildtype	8	15yo/F	AA	GBM	12	13	Midline (Thalamus)	Biopsy	NA (autopsy)	Yes	RT+ bevacizumab + TMZ, bevacizumab + TMZ + irinotecan
	9	18yo/M	GBM	GBM	25.3	25.3	Hemisphere	GTR	STR	Yes	RT, bevacizumab + irinotecan
	10	10yo/F	HGG Gr III w neuronal component	HGG Gr IV w neuronal component	9.3	17.3	Hemisphere	GTR	STR	Yes	RT, lapatinib + bevacizumab, VP16
	11	14yo/F	GBM	GBM	3.8	7.1	Hemisphere	Biopsy	NA (autopsy)	Yes	RT + TMZ, bevacizumab
	12	17yo/M	GBM	GBM	29.1	29.1	Hemisphere	STR	STR	No	RT, VP16, BMT
	13	19yo/M	AA	AA	39.2	39.2	Hemisphere	GTR	GTR	No	RT+ TMZ, TMZ
	14	12yo/M	GBM	GBM	13.8	13.8	Hemisphere	GTR	GTR	No	RT + TMZ, TMZ + lomustine
NF1 germline	15	15yo/M	AA	GBM	42.4	65.4	Hemisphere	GTR	NA (autopsy)	Yes	RT, cisplatin + cyclophosphamide +topotecan + vincristine, TMZ, bevacizumab, rapamycin, cabozantinib
	16	23yo/F	LGG Gr II w pilocytic features	GBM	9.5	9.5	Hemisphere	GTR	STR	Yes	Surgery

*Abbreviations*: *M* Male, *F* Female, *HGG* High Grade Glioma, *GBM* Glioblastoma, *Dx* Diagnosis, *AA* Anaplastic Astrocytoma, *GTR* Gross Total Resection, *NA* Not Available, *STR* Subtotal Resection, *RT* Radiation Therapy, *TMZ* Temozolomide, *VP16* Etoposide, *BMT* Bone Marrow Transplantation

*Targeted therapies:* Bevacizumab (Genentech, San Francisco, CA), vorinostat (Merck, Whitehouse Station, NJ), vandatenib (AstraZeneca, Wilmington, DE), dasatinib (Bristol-Myers Squibb, Princeton, NJ), lapatinib (Novartis, East Hanover, NJ), cabozantinib (Exelixis, San Francisco, CA)

### Core oncogenic mutations are shared in primary and recurrent pHGGs

**Fig. 1:**
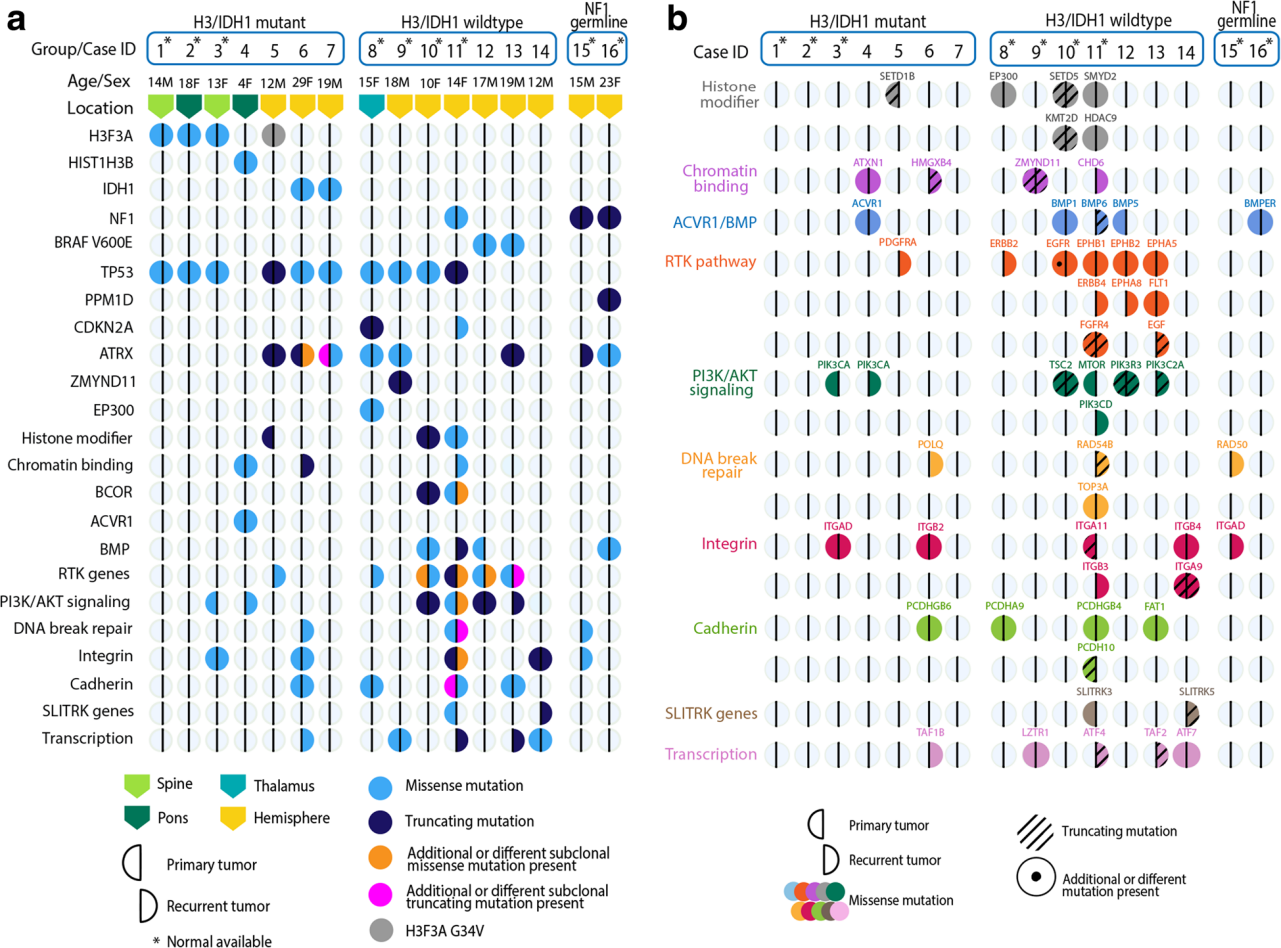
Mutational profile of primary and recurrence tumor pairs from 16 pediatric High-Grade Glioma (pHGG) patient samples analyzed in this study. Vertical columns of circles represent a tumor pair from an individual patient. The *left* half of a *circle* represents the primary tumor and the *right* half represents the recurrence. A fully colored circle indicates that both primary and recurrence tumors harbor the same mutation, and a half-colored *circle* indicates a mutation specific to the primary or recurrence. *Horizontal rows* show individual genes or gene groups/pathways that are mutated. Mutations shown are known to be pathogenic, found in COSMIC or TCGA, or have a high/medium functional impact. **a** Molecular group, age, sex and tumor location are indicated for each patient. Mutations are represented by colors: *light blue* = missense, *dark blue* = truncating (frameshift or nonsense), *orange* = additional/different missense, *pink* = additional or different truncating. **b** An expansion of each gene group showing individual genes mutated in each patient. Different gene groups are represented by different colors, truncating mutations are shown with a *slanted line*, and an individual *dot* represents an additional or different mutation is present

To determine the temporal stability of the mutational landscape of pHGGs, WES was performed on all 16 tumor pairs. Matched germline DNA was available for 9 patients. Samples were sequenced with an average coverage of 69X (range 3.5–200.4X) (Additional file 1: Table S1). Tumor pairs were stratified into three distinct molecular groups based on the identified mutation patterns (Fig. 1a, Additional file 2: Table S2). The first group was comprised of H3/IDH1 mutant tumors (7/16 pairs, 44%), where both the primary and recurrence harbored shared epigenetic driver mutations in *H3F3A* K27M (*n* = 3, HGG1, HGG2, HGG3), *HIST1H3B* K27M (*n* = 1, HGG4), *H3F3A* G34V (*n* = 1, HGG5), or *IDH1* R132H/S (*n* = 2, HGG6, HGG7). Consistent with previous studies, all H3K27M mutant tumors were in the brain midline (spine and pons), while *H3F3A* G34 V and *IDH1* mutant tumors were hemispheric [37, 41]. In addition, all six *H3F3A* and *IDH1* mutant tumors had co-occurring *TP53* mutations while the *HIST1H3B* (Histone 3.1) K27M mutant DIPG had a known associated *ACVR1* R258G mutation (HGG4) [14, 42, 51]. H3K27M/G34V and *IDH1* mutations were as expected clonal and conserved across primary and recurrent tumor pairs. Similarly, *ACVR1* and *TP53* mutations, which have been shown to be obligate partners in H3 and IDH mutagenesis, were also conserved throughout disease progression.

The second group had no identifiable H3 or IDH1 mutations, termed H3/IDH1 wildtype (7/16 pairs, 44%). This is a heterogeneous group of supratentorial, mostly hemispheric, tumors (6/7, 86%) with clonal mutations identified in *TP53* (4/7, 57%)*, ATRX* (3/7, 43%)*, BRAF* V600E (2/7, 29%), *BCOR* (1/7, 14%), *CDKN2A* (1/7, 14%), *ZMYND11* (1/7, 14%), and *EP300* (1/7, 14%) in tumor pairs (Fig. 1a). HGG8, the only H3/IDH1 thalamic wildtype tumor, had *TP53* missense, *ATRX* missense, *CDKN2A* nonsense, and interestingly, *EP300* missense mutations that were retained at recurrence. One sample (HGG11) had a hypermutated phenotype with 151 protein coding somatic mutations at diagnosis and 670 at relapse. The H3/IDH1 wildtype group was enriched for multiple mutations in receptor tyrosine kinase (RTK), phosphoinositide 3-kinase (PI3K) pathway, histone modifiers, integrin, and cadherin genes (Fig. 1a, b). Except for rare mutations, most of these genetic alterations were not present across tumor pairs, and seemed to occur more frequently at disease recurrence (Fig. 1a, b).

Lastly, the third molecular group was composed of patients with *NF1* germline truncating mutations (2/16, 12%) with associated HGG (HGG15, HGG16). There were few SNVs identified in the primary tumors from these 2 cases. The primary tumor of HGG15 had no detectable driver SNVs, however, it acquired an *ATRX* frameshift mutation as well as a *RAD50* missense mutation at recurrence (Fig. 1b). In HGG16, both primary and recurrent samples harbored *PPM1D* nonsense (L513X) and *ATRX* missense mutations. Previous studies have shown that *PPM1D* mutations affect p53 function and are mutually exclusive of *TP53* mutations [55]. This mutation pattern in the NF1 mutant group is reminiscent of G34R/V-*H3F3A* mutated HGGs which co-occur with *ATRX* and *TP53* alterations.

### Other mutational patterns at diagnosis and recurrence

In all groups of pHGG tumors, core oncogenic driver mutations in *H3 variants, IDH1, TP53, ACVR1, BRAF V600E,* and *PPM1D* were conserved at recurrence. As expected, the TP53 pathway (*TP53* or *PPM1D*) was the most frequently altered (11/16, 69%) across all tumor subgroups (Fig. 1a). *ATRX* mutations were also frequent (8/16, 50%) and, as previously described, were enriched in supratentorial samples (7/10, 70%) [1, 9, 14, 26]. In the H3/IDH1 mutant group, *ATRX* mutations were observed in 3 hemispheric tumor pairs: one pair with H3G34V (HGG5), and two with IDH1 R132H/S (HGG6, HGG7) mutations. Additionally, *ATRX* mutations were identified in three H3/IDH1 wildtype tumor pairs, two of which co-occurred with alterations in chromatin modifiers (HGG8, HGG9), and in both samples from patients with germline *NF1*. We observed the same *ATRX* mutation at recurrence in all tumor pairs of the H3/IDH1 wildtype subgroup. In *IDH1* mutant tumors, HGG7 harbored two *ATRX* alterations including a frameshift mutation exclusive to the primary tumor, while HGG6 acquired an additional *ATRX* missense mutation in the recurrence, in keeping with previous findings in IDH1 mutagenesis [19]. While taking into consideration the limitations imposed by our relatively small cohort, our findings indicate that in H3/IDH1 wildtype pHGGs, the same *ATRX* mutation, when present at diagnosis, is retained at disease recurrence despite gross total resection of the primary tumor in most cases. Interestingly, while loss of *BRAF* V600E at recurrence has been reported in adult gliomas [19], the two cases in our study (HGG12, HGG13) retained this mutation at recurrence.

### Novel epigenetic alterations in H3/IDH1 wildtype pHGGs

Two tumor pairs in the H3/IDH1 wildtype group showed potentially novel epigenetic drivers that converge to affect the same histone mark directly affected in pHGGs carrying H3.3 G34R/V or H3K27M mutations. Both primary and recurrent samples from HGG9, an adolescent patient with a parietal brain tumor, shared a *ZMYND11* frameshift mutation that possibly abrogates expression of this protein, and they also harbored concurrent *TP53* and *ATRX* missense mutations. These characteristics are typical of pHGGs that harbor H3.3G34R/V mutations. *ZMYND11/*BS69 has been shown to specifically recognize H3K36me3 to regulate and repress transcription [17, 49]. Conversely, H3.3G34R/V mutant nucleosomes affect the trimethylation of K36 on the H3.3 mutant nucleosomes [24], impairing the recognition of H3K36me3 ZMYND11/BS69 and its action on modulating translation [49]. In HGG8, both primary and recurrent thalamic tumors shared a mutation in *EP300* (D1339N), concurrent with *TP53* and *ATRX* missense mutations and a *CDKN2A* nonsense mutation. The *EP300* gene encodes a histone acetyltransferase (HAT), with acetylation activity at H3K27, and has been shown to regulate transcription through multiple mechanisms of chromatin remodeling [33]. The D1339N mutation has been identified in multiple tumor types, but not in HGG, and is presumed to affect the enzymatic activity of the protein [8, 25]. It is worth noting that HGG8 harboring mutant EP300 was a midline tumor, a location where H3K27M mutations known to affect H3K27 acetylation usually account for the vast majority of driver mutations. Taken together, these data suggest that both these novel epigenetic mutations are relevant in the setting of pHGGs, and may possibly mimic the effect of genetic drivers in H3-mutant tumors.

### Activating mutations of the RTK-PI3K pathway are not always conserved

Mutations in the RTK genes involving *EGFR, ERBB2, ERBB4, FLT1* and *EPHA/B*, were identified in 5/7 (71%) of the H3/IDH1 wildtype tumor pairs. Many of these were exclusive to the primary or recurrent sample (Fig. 1b). In the H3/IDH1 mutant tumor HGG5, a *PDGFRA* Y288C missense mutation was acquired at recurrence. Similar to previous reports, PI3K mutations were temporally heterogeneous [30], where 1/5 tumor (20%) exhibited a shared mutation in a PI3K regulatory subunit *PIK3R3* (HGG12). Additionally, subclonal mutations in the PI3K catalytic subunit were private to the primary tumor only in one case (HGG3) and were acquired at recurrence in two cases (HGG4, HGG13). Prior to our study, testing of a different tumor sampling from HGG3 was performed using a clinical genomic panel. This analysis identified a low frequency *PIK3CA* H1047R hotspot mutation that was not found in either the WES analysis of a different primary tumor tissue block or targeted high-depth sequencing of multiple samplings of the recurrent tumor (Additional file 3: Figure S1). In the hypermutated HGG11 tumor pair, the primary tumor harbored a missense *MTOR* mutation, while at recurrence the tumor acquired a PI3K catalytic subunit *PIK3CD* passenger mutation (Fig. 1b).

### Mutational burden, allelic imbalance and copy number variations

**Fig. 2:**
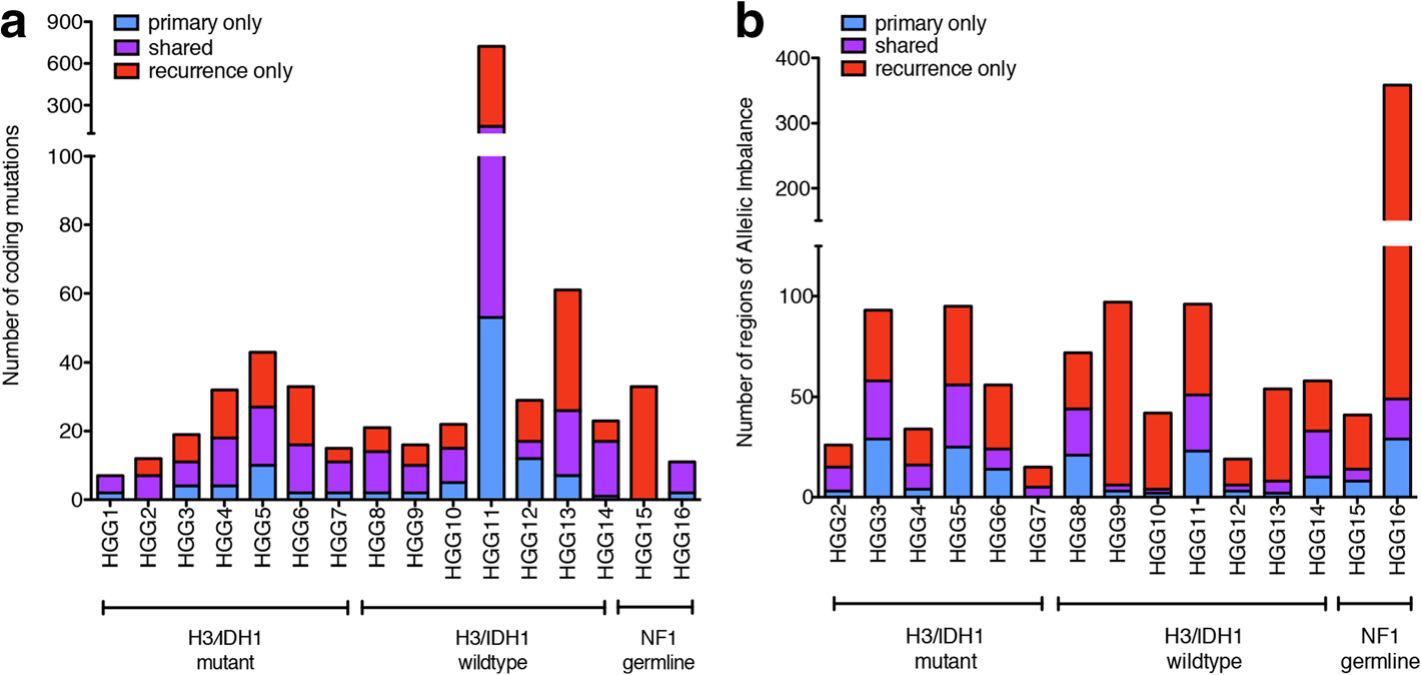
Number of mutations (**a**) or regions of allelic imbalance (**b**) calculated by ExomeAI [29] specific to the primary tumor (*blue*), recurrence (*red*), or shared (*purple*) in the pHGG tumor pairs analyzed in this study. See also Additional files 2 and 8: Tables S2 and S4

Analysis of the mutational burden showed no statistically significant difference in the number of mutations between primary and recurrent tumors across all groups (paired t-test, *p* = 0.24) (Fig. 2a, Additional file 4: Table S3, Additional file 5: Figure S2). It is worth noting that within the limitations of sample size, we observed a trend towards an increase in the mutational burden at recurrence that did not reach statistical significance despite the use of TMZ as adjuvant therapy in 10/16 (63%) pHGGs. In HGG11, we observed a marked increase in the number of somatic mutations in the primary (*n* = 151) and at recurrence (*n* = 670) compared to all other tumor samples, indicating a hypermutated phenotype. We identified and validated a germline *MLH1* splice missense mutation, and also performed immunohistochemistry on MMR proteins (MLH1, MSH6, MSH2, PMS2) on the primary HGG11 tumor (Additional file 6: Figure S3). Although IHC results did not show loss of any MMR proteins, we hypothesize that the splice mutation that translated extra inframe amino acids (data not shown), resulted in a dysfunctional yet nuclear-localized MLH1 protein. This may explain MMR IHC nuclear positivity in the setting of mismatch repair deficiency resulting in hypermutation. Interestingly, the mutation burden in that case dramatically increased at recurrence, which may be attributable to the combined effects of radiation and TMZ treatment [45].

To further assess chromosomal alterations in all of the primary-recurrent tumor pairs, we used WES data to analyze the state of allelic imbalance using ExomeAI [29]. Copy Number Variations (CNVs) were analyzed in eight tumor pairs with available matched normal. We calculated the numbers of allelic imbalance regions as shared or specific to the primary or recurrent tumor (Fig. 2b, Additional file 4: Table S3), regardless of the size of each region. Similar to mutation counts, there was no significant difference in the number of regions of allelic imbalance between the primary and recurrent tumors across all subgroups (paired t-test, *p* = 0.071). One tumor pair, HGG9, was particularly remarkable as there was an increased number of allelic imbalance regions in the recurrent tumor compared to the primary. Assessment of copy number variations confirmed genome-wide loss of heterozygosity events at recurrence resulting in a copy number neutral genome (Additional file 7: Figure S4), compatible with radiotherapy-induced chromosomal alterations [22, 54]. Both *NF1* germline cases also showed an increase in the number of regions of allelic imbalance. In both *NF1* germline recurrent tumors, CNV analysis showed loss of heterozygosity in the whole q arm of chromosome 17, containing the *NF1* gene locus (Additional files 8 and 9: Tables S4 and S5). Our limited CNV analysis did not show any focal deletions or amplifications in genes of interest (*PDGFRA, MYC, CDKN2A/B, CDK6*) that have been previously implicated in pHGG [37]. In 5 of 6 tumors with *TP53* mutations, large copy number alterations in the p arm of chromosome 7 were present that included the *TP53* gene loci (Additional file 8: Table S4).

### DNA methylation subgroup is maintained at recurrence in pHGG

**Fig. 3:**
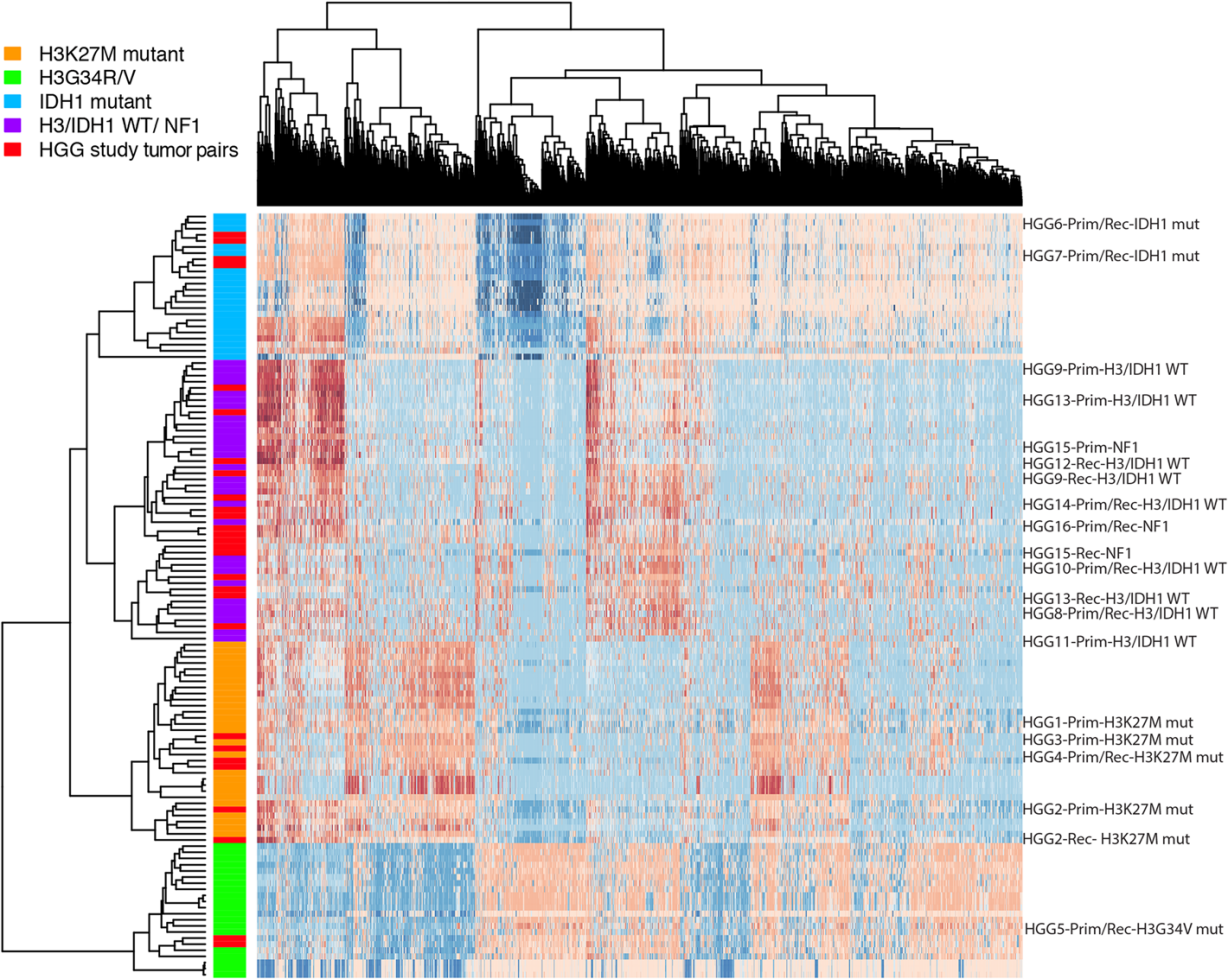
Methylation heatmap of pHGG tumors analyzed in this study. Primary and/or recurrent tumors from 16 pHGG samples from this study were analyzed with a large in-house dataset using 450 K methylation probes for clustering. In specific cases, tumor DNA from the primary only (HGG1, HGG3, HGG11) and recurrence only (HGG12) were available. *Red bars* within the colored group on the left represent the clustering of pHGG tumor samples within the known pHGG molecular group. Methylation groups are represented by colors: *orange* = H3K27M, *green* = H3G34R/V, *blue* = IDH1 mutant, *purple* = histone wildtype (WT). HGG case IDs for each tumor are indicated on the right. The label Prim/Rec indicates the clustering of both the primary and recurrence together. When the clustering location is different for the primary and recurrence tumor, the label is indicated as Prim (Primary) or Rec (Recurrence)

We performed unsupervised clustering of DNA methylation in tumor pairs integrated into a larger pHGG in-house data set (Fig. 3). As expected, tumors in the H3/IDH1 mutant group clustered within their respective methylation cluster, while H3/IDH1 wildtype and *NF1* germline groups clustered with H3/IDH1 wildtype HGGs. In all cases, the recurrent tumors clustered within the same methylation subgroup, similar to findings in other brain tumors including DIPG and medulloblastoma [30, 31].

## Discussion

In this work, we performed whole exome sequencing on 16 primary and recurrent pHGG pairs including two pHGGs from patients with germline *NF1* mutations, and provide insight into the temporal genomic evolution of these tumors. A direct comparison of the mutational landscape of paired samples reveals that oncogenic driver mutations are typically conserved. The identification of these mutations in both the primary and recurrent tumors suggests that these mutations are early initiating events in tumorigenesis, are stable, and unaffected by treatment. This is in contrast to adult GBM where cancer driver mutations can be subclonal in the primary and recurrent tumors [19].

In our dataset, 10 of 16 patients were treated with TMZ, and despite a trend towards an increase in the number of mutations at recurrence, there was no statistically significant increase in mutational burden. This is in contrast to adult GBM, where an increase in mutational burden is observed with TMZ treatment [19]. Although our sample size is small, our findings may reflect a different biological process in response to TMZ in children compared to adults and warrants further evaluation. We observed one H3/IDH1 wildtype primary tumor (HGG11) with an increased number of somatic mutations compared to other primary tumors, and was identified to harbor a germline *MLH1* splice missense mutation. Immunohistochemical analysis did not show loss of the MMR proteins, however, we hypothesize that the missense splice mutation likely resulted in the translation of a dysfunctional MLH1 protein product to cause mismatch repair deficiency (MMRD) and hypermutation. After treatment with radiation and TMZ, this tumor acquired an increased number of somatic mutations compared to the primary tumor, suggesting that treatment further exacerbated the hypermutated phenotype. Several controversial and contradictory studies have variably reported the presence of microsatellite instability which results in mismatch repair deficiency in pediatric HGG and adults [10, 44], highlighting the need for further studies. Future genetic testing for MMRD in pediatric HGG patients could steer treatment towards immunotherapy, as immune checkpoint blockade has shown clinical benefits in MMRD colorectal cancers as well as children with high-grade glioma [4, 23].

Similar to findings in adult *IDH1*-mutant gliomas [19], we identify heterogeneous *ATRX* alterations among *IDH1* mutant pHGG tumor pairs. While *IDH1* mutant tumors are more common in adult GBM and occur in up to 98% of secondary GBMs, they make up less than 10% of all pediatric HGGs [2, 52]. In contrast to *IDH1*-mutant gliomas, *ATRX* mutations associated with H3G34V, *ZMYND11*, *EP300*, or *BRAF* V600E were stable across the disease course in our study. Additionally, the *BRAF* V600E mutation was present in both primary and relapse samples in two children in our study which is in contrast to adult studies where it was identified either at diagnosis or at recurrence [19].

H3/IDH1 wildtype pHGGs have previously been shown to be a diverse group of tumors with mutations in many cancer pathways [35, 37, 51], but have not been directly linked to any particular epigenetic driver as is the case with H3 and IDH1 mutant tumors. Our data reflect the heterogeneity of tumors in the H3/IDH1 wildtype group while also identifying two novel pHGG epigenetic cancer drivers (*ZMYND11* and *EP300*) in this group. ZMYND11 has recently been described as an epigenetic regulator that specifically interacts with H3K36me3 to regulate transcription. Wen et al. have reported that H3 G34R/V mutations impair binding of ZMYND11 to an H3.3K36me3 peptide, suggesting that H3.3 G34R/V and *ZMYND11* mutations alter H3K36me3 levels in similar fashions [49]. To the best of our knowledge, *ZMYND11* mutations have not been previously described in pHGGs. The tumor harboring this mutation (HGG9) was located in the right parietal lobe and carried partner mutations in *ATRX* and *TP53*, further supporting its similarity to hemispheric H3.3 G34R/V mutated tumors. In addition, inactivating mutations identified in the HAT gene *EP300* have been implicated in a wide array of cancer types including diffuse large B cell lymphoma [34], head and neck, esophageal, colorectal, medulloblastoma and non-small cell lung carcinoma [7, 15]. We also report a specific *EP300* hotspot D1399N mutation (HGG8) which has not been previously identified in HGGs. Structural analysis of EP300 has shown that the D1399 residue has effects on the conformation of the HAT domain, specifically the L1 loop [25]. This is also an inactivating mutation which abolishes autoacetylation required for HAT activity, thus affecting post-translational modification of K27 on H3 variants [8]. Interestingly, *EP300* D1399Y mutations alter its interaction with transcription factor AP-2alpha indirectly leading to the transactivation of Myc [16]. Moreover, the tumor harboring the *EP300* mutation was located in the thalamus which is a neuroanatomical structure in the brain midline where the majority of HGGs harbor H3K27M mutations. This novel epigenetic mutation may reproduce some of the effects of K27M in a wildtype H3K27 tumor. In our study, the tumor with the *EP300* D1399N mutation had increased Myc expression (data not shown), suggesting that this particular EP300 mutation may also play a role in Myc-related oncogenesis similar to K27M mutagenesis. Although interesting, these findings need further testing and functional validation in relevant disease models. The two HGGs from patients with germline NF1 did not show a high mutational burden at diagnosis or at recurrence, and no clear associated driver mutation. Interestingly, a tendency towards increased copy number alteration was observed in both pairs at recurrence. These findings also need further validation on a larger sample set.

Somatic mutations in RTKs are common in adult GBM [5, 6] and are generally found at low frequencies in pHGGs [41]. Similar to our previous report [41], the H3/IDH1 wildtype group in this study seemed enriched with RTK mutations (5/7, 71%). One striking finding in this molecular group was the discovery of *EGFR* missense mutations in the primary occurrence of HGG10 (T790M and E709A), which were lost in the recurrence. A shared EGFR R222C missense mutation was present in both the primary and recurrent tumors, indicating that alteration of the RTK pathway is nonetheless conserved in the recurrent tumor. The *EGFR* T790M mutation has been implicated in acquired resistance to most EGFR tyrosine kinase inhibitors [21, 27]. This may, in part, explain tumor progression in this patient despite treatment with lapatinib (Novartis, East Hanover, NJ), and highlights the importance of identifying resistance-promoting mutations in the clinical setting. We also identified three tumors with targetable RTK lesions (*PDGFRA, ERBB2, ERBB4*) that were exclusive to the recurrent tumor (HGG5, HG8, HGG11), indicating that genomic data from tumor tissue at recurrence may provide better guidance for therapeutic choices. Conversely, one case harbored a low level subclonal *PIK3CA* mutation that was discovered by a clinical genomics panel in the primary tumor, but was not identified by WES in different primary tumor blocks from the same case, nor in multiple samplings of the recurrent tumor. Excluding the subclonal nature of this mutation, and confirming its maintenance at recurrence are important therapeutic considerations before embarking on targeted treatment, especially with single agents such as rapamycin used in this patient.

## Conclusions

In conclusion, this study further highlights the molecular distinction between pediatric and adult HGGs, especially in therapy-induced tumor evolution. We show that genes with driver mutations (H3, *TP53, PPMID, ZMYND11, EP300*) as well as some targetable mutations (e.g. *IDH1*, *BRAF* V600E) are conserved. Importantly, we demonstrate that some actionable mutations are unstable (*PI3K, EGFR*), indicating that re-biopsy is warranted in order to optimize personalized therapy. The presence of subclonal targetable alterations concurrently with driver mutations supports the use of combination therapy approaches to address disease biology and evolution with the aim of improving patient outcomes.

## Additional files

Additional file 1: Table S1. Tissue location and type, tumor content, WES coverage and digital droplet PCR results in 16 pairs of pHGG analyzed in this study. (XLSX 13 kb)

Additional file 2: Table S2. Somatic and putative somatic mutation information for 16 pairs of pHGGs analyzed in this study. (XLSX 301 kb)

Additional file 3: Figure S1. IGV views a subclonal low frequency *PIK3CA* mutation in HGG3 from a clinical sequencing panel, WES, and targeted sequencing. (PDF 2380 kb)

Additional file 4: Table S3. Number of Single Nucleotide Variants (SNVs) and regions of Allelic Imbalance (AI) present in tumors as shared, primary only, or recurrence only in the pHGG tumor pairs analyzed in this study. (XLSX 13 kb)

Additional file 5: Figure S2. Percentages of SNVs and regions of Allelic Imbalance as shared, primary only and recurrence only. (PDF 908 kb)

Additional file 6: Figure S3. Immunohistochemical staining for the MMR panel (MLH1, MSH2, MSH6 and PMS2) in the HGG11 primary tumor. (PDF 23521 kb)

Additional file 7: Figure S4. Genome-wide view of copy number variations in HGG9 primary and recurrence tumors calculated from Whole Exome Sequencing data (PDF 2757 kb)

Additional file 8: Table S4. Chromosomal location of AI segments in 15 pairs of pHGG analyzed in this study. (XLSX 61 kb)

Additional file 9: Table S5. Copy number variation (CNV) segments in primary and recurrence tumors from 8 of 16 pairs of pHGG with matched normal tissue available. (XLSX 71 kb)

## Abbreviations

IDH1: isocitrate dehydrogenase 1
TP53: tumor protein 53
ACVR1: activin a receptor, type I
ZMYND11: zinc finger MYND domain-containing protein 11
EP300: histone acetyltransferase p300
BRAF: b-raf proto-oncogene
NF-1: neurofibromatosis 1
ATRX: alpha-thalassemia/mental retardation syndrome, Nondeletion type, x-linked
EGFR: epidermal growth factor receptor
ERBB2: Erb-B2 receptor tyrosine kinase 2
PDGFRA: platelet derived growth factor receptor alpha
PI3K: phosphoinositide 3-kinase
