# Supplementary material for: Characterizing temporal genomic heterogeneity in pediatric high-grade gliomas
Source: Acta Neuropathol Commun. 2017 Oct 30;5:78. doi: 10.1186/s40478-017-0479-8 (PMC5663045; doi:10.1186/s40478-017-0479-8)

Supplementary Figure S1. IGV view of sequencing results from HGG3 primary (P) and recurrence (R) showing a subclonal mutation in *PIK3CA* H1047R

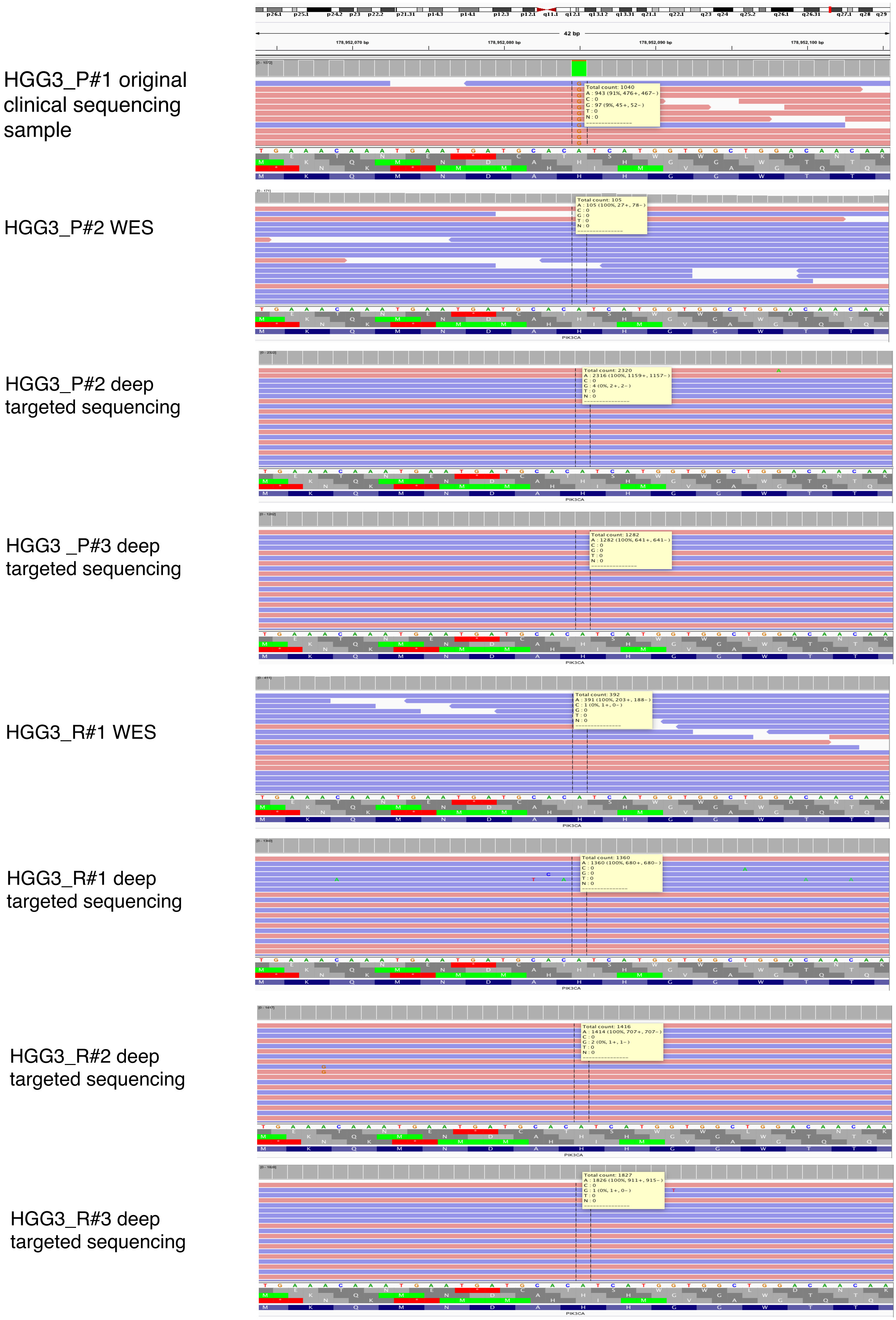

Supplement: Supplementary file 3 — IGV views a subclonal low frequency PIK3CA mutation in HGG3 from a clinical sequencing panel, WES, and targeted sequencing. (PDF 2380 kb) [file 40478_2017_479_MOESM3_ESM.pdf]
