# Supplementary material for: Characterizing temporal genomic heterogeneity in pediatric high-grade gliomas
Source: Acta Neuropathol Commun. 2017 Oct 30;5:78. doi: 10.1186/s40478-017-0479-8 (PMC5663045; doi:10.1186/s40478-017-0479-8)

Supplementary Figure S2: Percentages of SNVs and regions of Allelic Imbalance as shared, primary only and recurrence only.

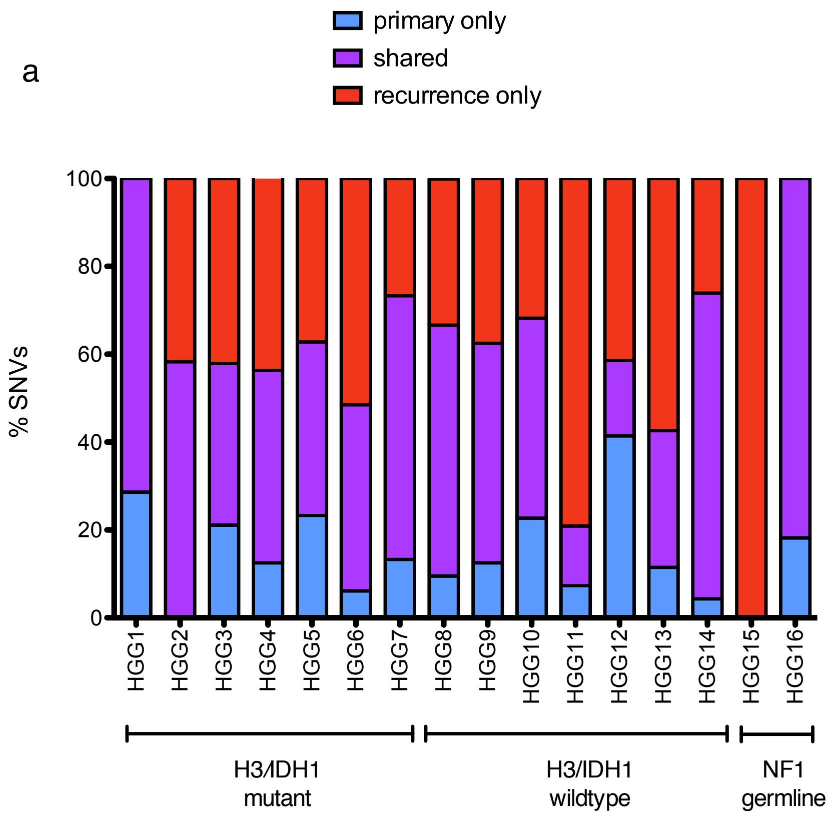

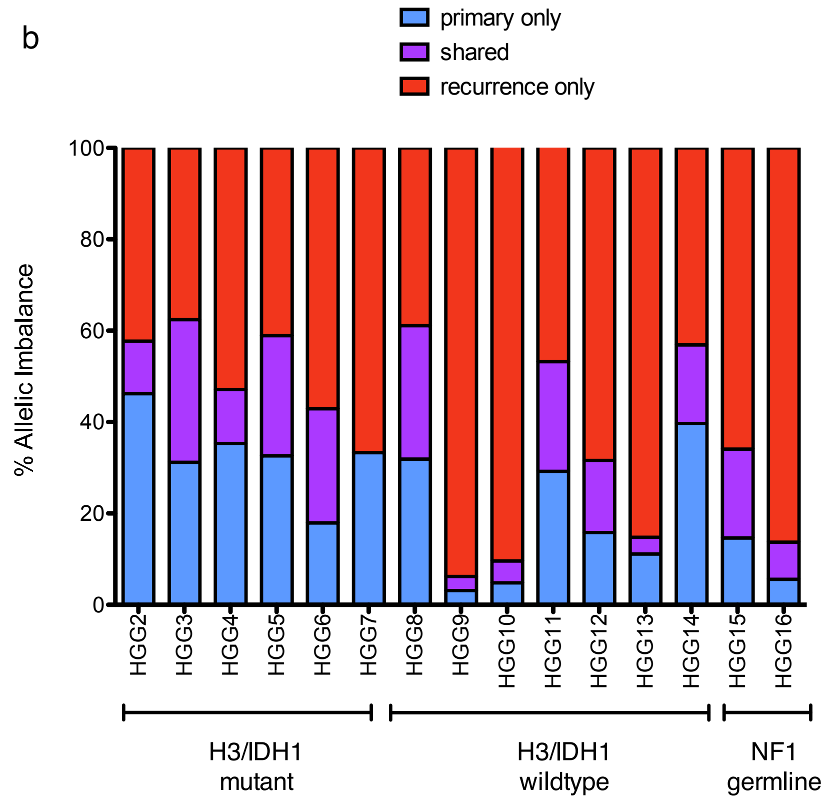

Supplement: Supplementary file 5 — Percentages of SNVs and regions of Allelic Imbalance as shared, primary only and recurrence only. (PDF 908 kb) [file 40478_2017_479_MOESM5_ESM.pdf]
