# Supplementary material for: Characterizing temporal genomic heterogeneity in pediatric high-grade gliomas
Source: Acta Neuropathol Commun. 2017 Oct 30;5:78. doi: 10.1186/s40478-017-0479-8 (PMC5663045; doi:10.1186/s40478-017-0479-8)

Supplementary Figure S3. Immunohistochemical staining for the MMR panel (MLH1, MSH2, MSH6, PMS2) in the HGG11 primary tumor.

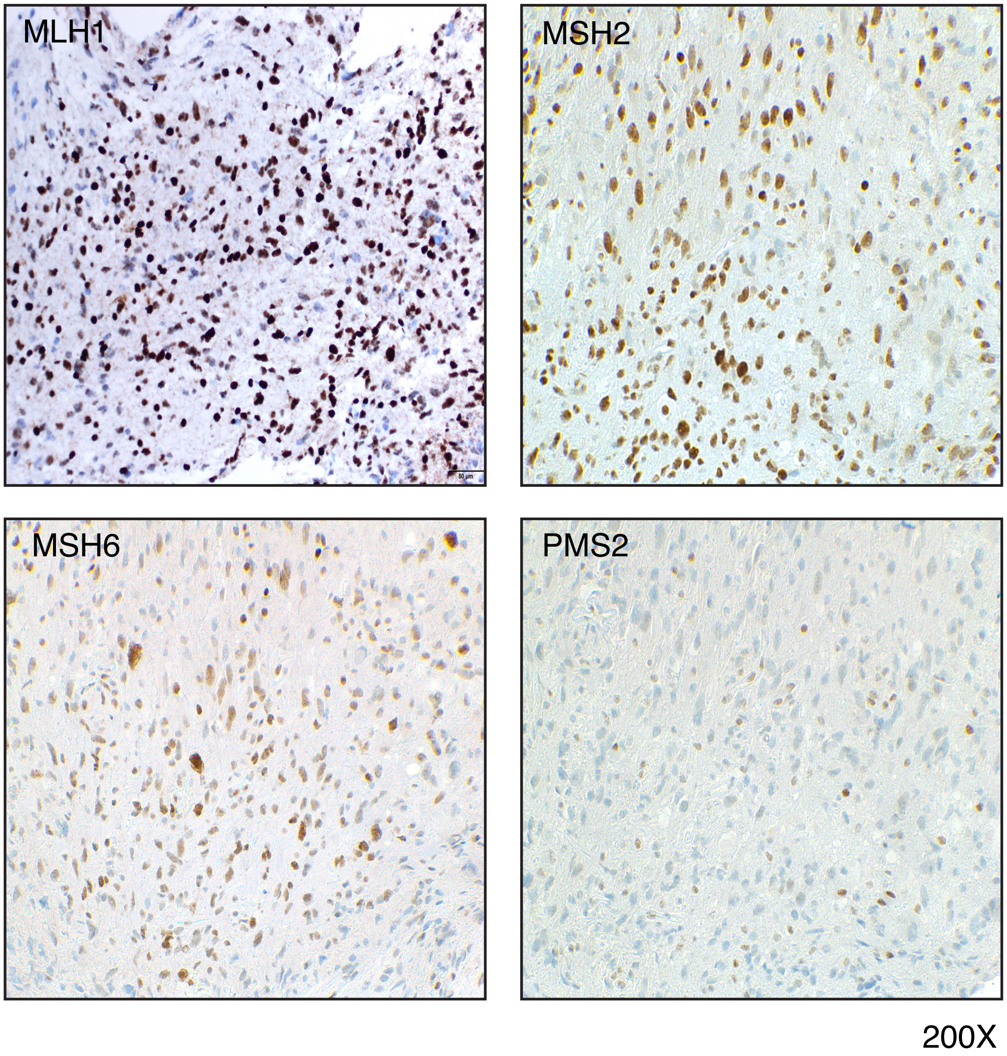

Supplement: Supplementary file 6 — Immunohistochemical staining for the MMR panel (MLH1, MSH2, MSH6 and PMS2) in the HGG11 primary tumor. (PDF 23521 kb) [file 40478_2017_479_MOESM6_ESM.pdf]
