# Supplementary material for: Characterizing temporal genomic heterogeneity in pediatric high-grade gliomas
Source: Acta Neuropathol Commun. 2017 Oct 30;5:78. doi: 10.1186/s40478-017-0479-8 (PMC5663045; doi:10.1186/s40478-017-0479-8)

**Supplementary Figure S4.** Genome-wide view of copy number variations in HGG9 primary and recurrence tumors calculated from Whole Exome Sequencing data

## HGG9-primary

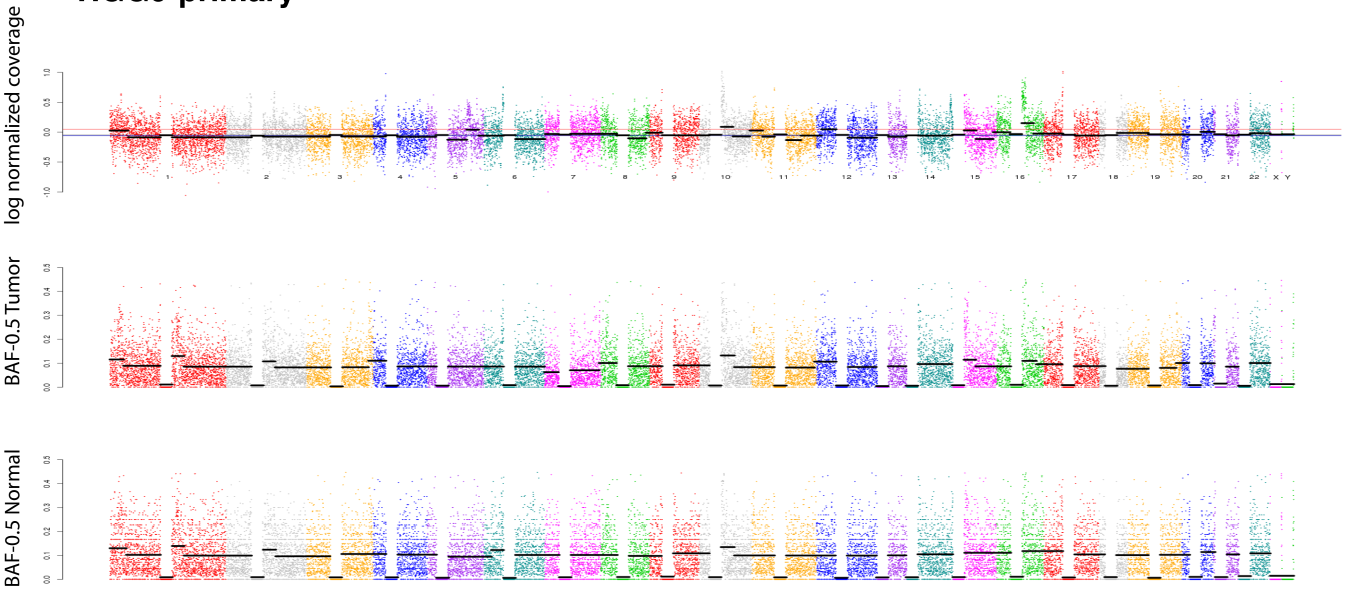


## HGG9-recurrence

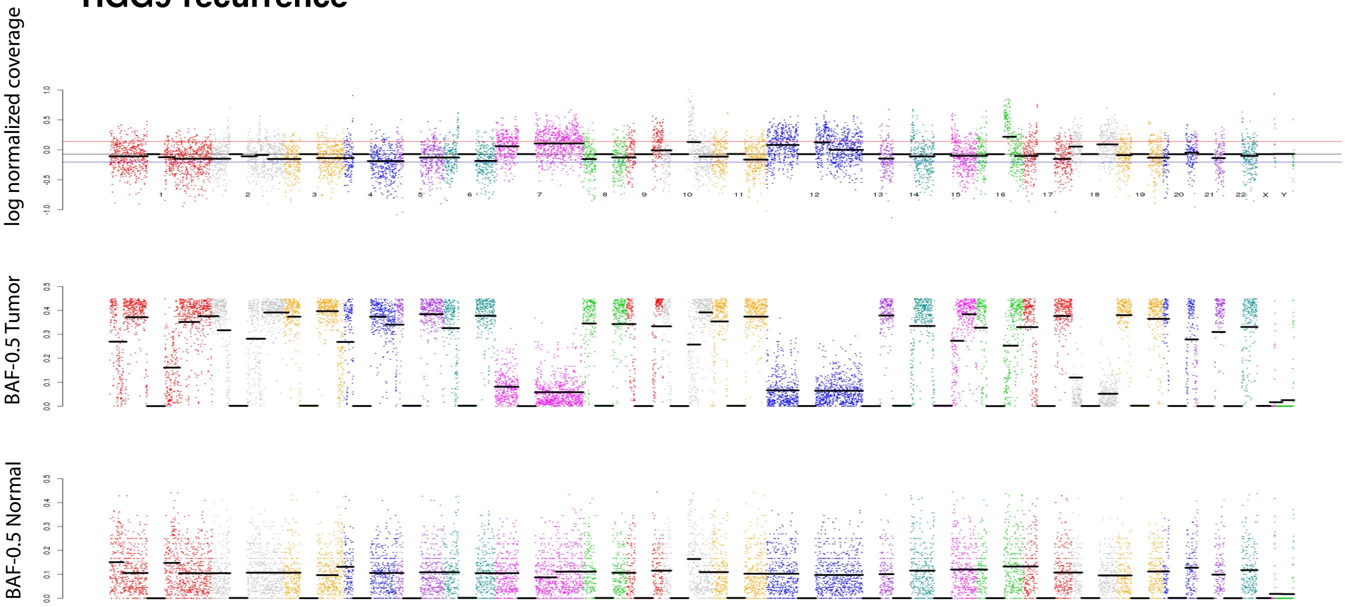

Supplement: Supplementary file 7 — Genome-wide view of copy number variations in HGG9 primary and recurrence tumors calculated from Whole Exome Sequencing data (PDF 2757 kb) [file 40478_2017_479_MOESM7_ESM.pdf]
